# Ultra High Performance Liquid Chromatography Method for the Determination of Two Recently FDA Approved TKIs in Human Plasma Using Diode Array Detection

**DOI:** 10.1155/2015/215128

**Published:** 2015-05-25

**Authors:** Marwa Fouad, Maxime Helvenstein, Bertrand Blankert

**Affiliations:** ^1^Pharmaceutical Chemistry Department, Faculty of Pharmacy, Cairo University, Kasr El-Aini Street, Cairo 11562, Egypt; ^2^Laboratory of Pharmaceutical Analysis, Faculty of Medicine and Pharmacy, Research Institute for Health Sciences and Technology, University of Mons (UMONS), Place du Parc 20, 7000 Mons, Belgium

## Abstract

Generally, tyrosine kinase inhibitors have narrow therapeutic window and large interpatient variability compared to intrapatient variability. In order to support its therapeutic drug monitoring, two fast and accurate methods were developed for the determination of recently FDA approved anticancer tyrosine kinase inhibitors, afatinib and ibrutinib, in human plasma using ultra high performance liquid chromatography coupled to PDA detection. Diclofenac sodium was used as internal standard. The chromatographic separation was achieved on an Acquity UPLC BEH C18 analytical column using a mobile phase combining ammonium formate buffer and acetonitrile at a constant flow rate of 0.4 mL/min using gradient elution mode. A *µ*SPE (solid phase extraction) procedure, using Oasis MCX *µ*Elution plates, was processed and it gave satisfying and reproducible results in terms of extraction yields. Additionally, the methods were successfully validated using the accuracy profiles approach (*β* = 95% and acceptance limits = ±15%) over the ranges 5–250 ng/mL for afatinib and from 5 to 400 ng/mL for ibrutinib in human plasma.

## 1. Introduction

In recent years, multiple tyrosine kinase inhibitors (TKIs) have been approved as monotherapy for cancer treatment. These targeted anticancer compounds are directed against tyrosine kinases, which play an essential role in the transduction of growth signals in cells [1]. Since they specifically inhibit cellular processes that are deregulated in various types of tumor cells, they were initially considered to be less toxic than conventional chemotherapy. However, it appears that, similar to conventional chemotherapy, dose interruptions or reductions due to adverse effects are necessary in a large number of patients which indicates that TKIs have a narrow therapeutic window [2–5]. Additionally, high pharmacokinetic variability (both interpatient and intrapatient) in plasma levels was found, which results in highly variable plasma concentrations and consequently drug-exposure. This suggests that plasma levels may be more predictive than absolute dose in predicting treatment response and adverse effects [6–11]. TKIs have most of the characteristics that are required for therapeutic drug monitoring (TDM), such as a narrow therapeutic window, large interpatient variability compared to intrapatient variability, and the chronic use until disease progression [12]. Therefore, TDM might be a very promising tool for this new class of drugs in order to improve treatment benefit by reducing drug toxicity, reducing drug resistance, and increasing efficacy. Moreover, rational quantification of TKI plasma levels can provide a better understanding of treatment failure or suboptimal response in patients receiving TKIs [13]. Thus, to support clinical pharmacological studies and to address observations in daily clinical practice, it was essential to develop and validate a quantitative bioanalytical assay to quantify TKIs in plasma.

Afatinib (AFA) is an orally administered, irreversible tyrosine kinase inhibitor of the ErbB family of receptors and it is reported to be a potential treatment for a variety of solid tumors. It is potent and highly selective as it irreversibly inhibits signaling from all ErbB family dimers: ErbB1, ErbB2, ErbB3, and ErbB4 [14–16]. As these receptors are involved in cell proliferation, differentiation, and apoptosis, their inhibition may play a critical role in the prevention of tumor growth and spread, including epidermal growth factor receptor- (EGFR-) mutation-positive non-small cell lung cancer (NSCLC) and metastatic head and neck cancer [17–21].

Ibrutinib (IBR) irreversibly inhibits Bruton's tyrosine kinase (BTK), an enzyme responsible for proliferation, differentiation, apoptosis, and cell migration of B-cells, by binding to a cysteine residue (Cys-481) in the BTK active site [22, 23]. Because constitutive activation of B-cell receptor signaling is important for survival of malignant B-cells, BTK inhibition results in decreased malignant B-cell proliferation and survival. Nonclinical studies show that IBR also inhibits B-cell migration and substrate adhesion in vitro. It is proposed for the treatment of patients with relapsed or refractory mantle cell lymphoma (MCL) and chronic lymphocytic leukemia (CLL). It is only approved for use in patients who have received at least one prior therapy, limiting its use as a potential frontline therapy [24, 25].

A publication from Stopfer et al. in 2012 describes a HPLC-MS/MS method for AFA pharmacokinetics assessment, but without considering a real validation procedure from the analytical point of view [26]. It is why the present work, to the best of our knowledge, proposes the first fully validated methods for the quantification of two recently FDA approved TKIs [27–30], AFA and IBR, in biological matrix. Thus, the aim of our study was the development and validation of simple, sensitive, rapid, and reliable UPLC-DAD methods, suitable for the quantification of AFA and IBR in human plasma. The validation results herein of the suggested inexpensive method for the assay of these drugs can be broadly applicable to clinical routine and efficient for a wider panel of bioclinical laboratories. The described validations were performed according to accuracy profile strategy (*β*-expectation tolerance interval).

## 2. Materials and Methods

### 2.1. Reagents

**Figure 1:**
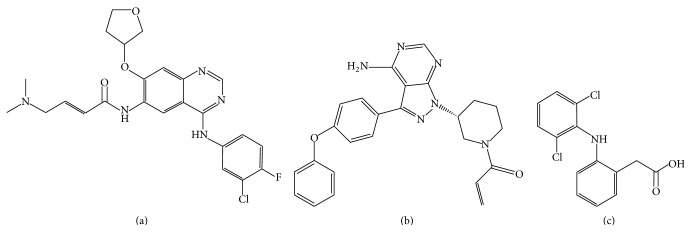
Chemical structures of (a) afatinib, (b) ibrutinib, and (c) diclofenac.

Afatinib, [N-[4-[(3-chloro-4-fluorophenyl)amino]-7-[[(3S)-tetrahydro-3-furanyl]oxy]-6-quinazolinyl]-4(dimethylamino)-2-butenamide] (Figure 1(a)), and Ibrutinib, [1-[(3R)-3-[4-Amino-3-(4-phenoxyphenyl)-1H-pyrazolo[3,4-d]pyrimidin-1-yl]piperidin-1-yl]prop-2-en-1-one] (Figure 1(b)), were purchased from Selleck Chemicals (Houston, TX, USA). Diclofenac sodium [2-(2-(2,6-dichlorophenylamino)phenyl)acetic acid sodium salt] (Figure 1(c)) came from ABC Chemicals (Wauthier-Braine, Belgium).

Phosphoric acid and methanol (HPLC grade) came from ChemLab (Zedelgem, Belgium). Ammonium hydroxide came from VWR (Fontenay-sous-Bois, France). Ammonium formate was purchased from Sigma-Aldrich (St. Louis, MO, USA). Formic acid and acetonitrile (UPLC grade) came from Biosolve (Valkenswaard, Netherlands). Ultrapure water (18.2 MΩ cm) was obtained with a Reference A+ Milli Q water purification system (Millipore, Brussels, Belgium).

### 2.2. Instrumental Conditions

Chromatographic analyses were performed on a Waters Acquity H-Class UPLC System (MA, USA) equipped with an Acquity UPLC BEH C18 (1.7 *μ*m; 2.1 mm × 50 mm) as analytical column, maintained at 40°C. Sample temperature was kept at 4°C. An optimized gradient elution was carried out at a constant flow rate of 0.4 mL/min. The mobile phase combines ammonium formate buffer (4 mM, pH 3.2 adjusted with formic acid) and acetonitrile (ACN). For AFA, the percentage of ACN was initially set at 5% and regularly increased up to 22% within 1 min. Thereafter, it was gradually increased to 80% until 2.7 min, kept constant until 2.9 min, and quickly decreased to 5% at 3.0 min. For IBR, the percentage of ACN was initially set at 5% and regularly increased up to 37% within 1 min. Thereafter, it was gradually increased to 90% until 2.7 min, kept constant until 2.9 min, and quickly decreased to 5% at 3.0 min. The total run time was 4.0 min and the injection volume was fixed at 10 *μ*L. The DAD detector was set at 268 nm. All data acquisition and chromatograms analyses were performed using Empower Software 3.0 (Waters).

### 2.3. Stock and Standard Solutions

Stock solutions of AFA (0.5 mg/mL), IBR (0.5 mg/mL), and diclofenac sodium internal standard (IS) (0.1 mg/mL) were made in methanol. Working solutions (40 *μ*g/mL for AFA and IBR, 10 *μ*g/mL for IS) were prepared by diluting the adequate volume in methanol. To prepare the standard solutions, working solutions of AFA and IBR were serially diluted with methanol to obtain the desired concentrations: 0.2–10 *μ*g/mL for AFA and 0.2–16 *μ*g/mL for IBR. Then, equal volumes of IS working solution and each of AFA and IBR standard solutions were mixed.

### 2.4. Sample Preparation

For the development and validation steps, plasma was thawed at room temperature and centrifuged at about 1920 RCF at 4°C for 10 min. Samples were prepared by spiking 100 *μ*L of plasma with 5 *μ*L prepared solution mixture of the target analyte and internal standard and diluted with 900 *μ*L or 100 *μ*L purified water for SPE or *μ*SPE, respectively. Finally, 20 *μ*L phosphoric acid was added and samples were vortex mixed for 15 seconds.

### 2.5. Solid Phase Extraction (SPE)

Sample solutions were applied to OASIS MCX cartridges which had previously been conditioned twice with 1 mL methanol and equilibrated with 1 mL purified water, successively. The cartridge was washed with 1 mL 2% formic acid in water (v/v). Elution was carried out by 1 mL elution mixture: acetonitrile/methanol/ammonium hydroxide (57 : 38 : 5), and the eluate was collected and evaporated to dryness using gentle stream of nitrogen. The residue was reconstituted with 100 *μ*L methanol.

For the validation procedure, Oasis MCX *μ*Elution Plates (30 *μ*M, Waters) were used with an extraction plate manifold (Waters, Milford, MA, USA). Each well of the plate was conditioned two times with 200 *μ*L of methanol and equilibrated with 100 *μ*L of ultrapure water. The samples (prepared as explained in Section 2.4) were loaded in each well. Formic acid (2%, v/v in water) washes were then performed. Elution was done in collection plates with 50 *μ*L of elution mixture, acetonitrile/methanol/ammonium hydroxide (57 : 38 : 5), and quickly diluted with 50 *μ*L of formic acid 4% (v/v) in water. During the validation step, recoveries at all the levels of concentration were calculated on three consecutive days. Areas of the peaks after MCX extraction were compared to those of standard solutions.

### 2.6. Validation Methodology

The developed UPLC method was validated using accuracy profiles concept. The Société Française des Sciences et Techniques Pharmaceutiques (SFSTP) commissions elaborated validation guidelines to help scientists to apply harmonized regulatory recommendations and to validate their analytical and biopharmaceutical procedures [31–33]. Their novel validation strategy was based on the total error (bias + standard deviation) and the accuracy profiles decision tool. Currently, this new protocol of validation becomes more attractive and knows a wider spreading among the scientific community [34–40].

Briefly, a procedure can be qualified as acceptable if the difference between every measurement (*x*) of a sample and its “true value” (*μT*) is inside the acceptance limits *λ* (predefined by the analyst depending to the objective of the method). The probability that the results will be in these acceptance limits should be superior or equal to a probability *β*. It can be translated into the following [31–33]:

(1) $$\begin{array}{c} P\left(\;\left|\;x-\mu T\;\right|<\lambda \;\right)\geq \beta . \end{array}$$

For each analyte, 6 calibration standards (CS) and 6 validation standards (VS) are realized in human plasma. The concentration range for CS and VS for AFA and IBR in plasma ranged from 5 to 250 ng/mL and from 5 to 400 ng/mL, respectively. The concentration of IS was 250 ng/mL. Each CS and VS was prepared on three consecutive days and analyzed each day in triplicate for CS and four times for VS. *β*-expectation tolerance intervals were computed for each analyte using *β* set at 95%. All data were computed with Excel software (Microsoft, USA). Accuracy profiles were drawn using acceptance limits at ±15% for the concentration range.

## 3. Results and Discussion

### 3.1. Developing the *μ*SPE and UPLC Method

During the optimization cycle, several chromatographic conditions were attempted using Acquity UPLC BEH C18 (1.7 *μ*m; 2.1 mm × 50 mm). Various mobile phase compositions like ACN with 0.1% formic acid and ACN with ammonium formate buffer (4 mM, pH 3.2) in different proportions were tried in an isocratic and gradient mode (data not shown). The final composition chosen is a mixture of ACN and ammonium formate buffer (4 mM, pH 3.2) for both compounds using a gradient elution mode. The chromatographic separation performed under these conditions is efficient and gives rise to well-shaped peaks with a rapid time analysis shorter than 4 min.

Selecting a proper detection wavelength is of great importance to ensure precise detection of the analytes and to achieve the goal of maximizing absorption and minimizing interference. The UV-Vis spectra acquired with the DAD detector exhibited a major absorption band at 258 for AFA and IBR but 268 nm was chosen as the wavelength of detection because it was found that minimum interferences have arisen from plasma components at this wavelength while keeping reasonable peak areas.

**Figure 2:**
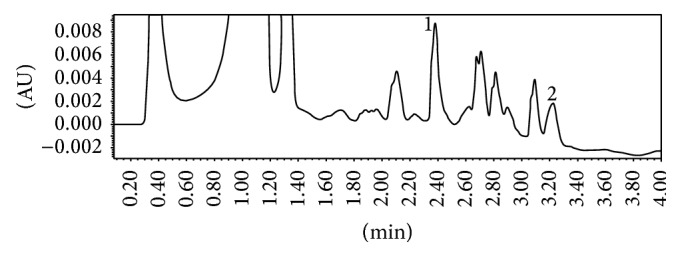
Typical chromatogram of human plasma sample spiked with (1) afatinib at 250 ng/mL (RT: 2.38 min) and (2) diclofenac at 250 ng/mL (RT: 3.22 min).

**Figure 3:**
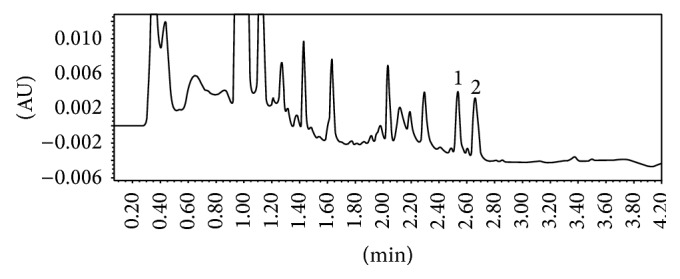
Typical chromatogram of human plasma sample spiked with (1) ibrutinib at 200 ng/mL (RT: 2.54 min) and (2) diclofenac at 250 ng/mL (RT: 2.66 min).

The chromatograms registered for AFA and IBR, in human plasma, show sharp and symmetrical peaks; the targeted compounds are separated with high resolution and selectivity, free from any interference (Figures 2 and 3).

The selected SPE stationary phase (MCX) is an ion exchange mode, specially designed for weakly basic compounds. Classical SPE MCX cartridges were only used in the preliminary phase to check if the MCX sorbent is the appropriate stationary phase for the extraction of target analytes and IS from human plasma. The selectivity of MCX stationary phase allowed a good resolution of the analyte from plasma matrix peaks. Consequently, Oasis MCX *μ*Elution plates were used for the validation procedure and it gave good chromatograms with well resolved peaks. *μ*Elution SPE presents the advantages to avoid any studied compounds degradation and no evaporation step is required which limits sample losses and saves time. Mean recoveries, with *μ*SPE MCX extraction, provided satisfying yields at 88.0 ± 2.2 and 93.0 ± 9.0 for AFA and IBR, respectively (see Table 1).

### 3.2. Validation

**Figure 4:**
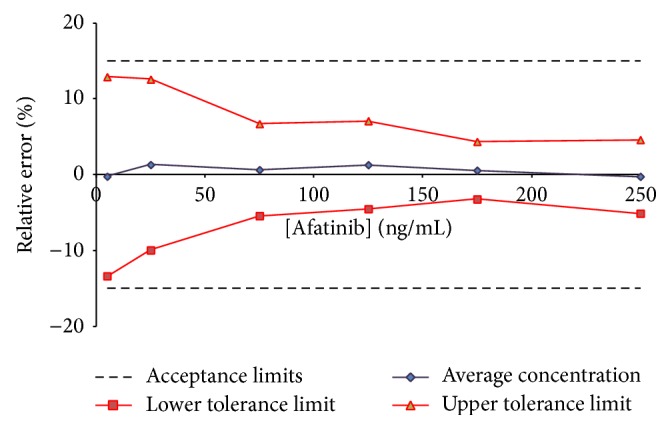
Accuracy profile for afatinib in human plasma; *β* expectation tolerance interval set at 95% and *λ* acceptance limits set at ±15%. All tested concentrations were validated (from 5 to 250 ng/mL).

**Figure 5:**
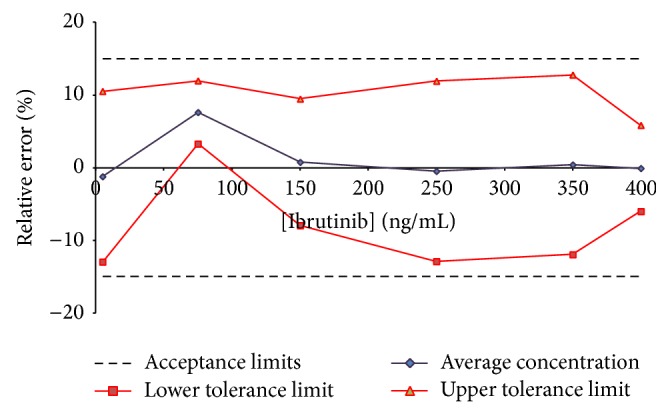
Accuracy profile for ibrutinib in human plasma; *β* expectation tolerance interval set at 95% and *λ* acceptance limits set at ±15%. All tested concentrations were validated (from 5 to 400 ng/mL).

Using the experimental data results from the validation procedure, we computed trueness (expressed in terms of relative bias (%)), precision (intermediate precision and repeatability), and accuracy. For each analyte, tolerance intervals limits were calculated (*β* = 95%) and accuracy profiles were drawn. The linear regression model allowed the validation of each analytical quantification method on the whole range of concentration (data of interest are summarized in Table 1). Figures 4 and 5 show accuracy profiles for each compound considered with linear regression model. As all tolerance intervals are comprised within the acceptance limits, this graphical tool permits to conclude to the validation of the developed quantification methods on the whole tested concentration ranges: from 5 to 250 ng/mL for AFA and from 5 to 400 ng/mL for IBR. The LLOQ on plasma assays for AFA and IBR are presented both to be of 5 ng/mL.

## 4. Conclusion

We have developed and validated two fast UPLC methods coupled to DAD for the quantitative analysis of two tyrosine kinase inhibitors (afatinib and ibrutinib) in human plasma. Human plasma spiked with these TKIs was successfully extracted using Oasis MCX 96-well *μ*Elution Plates. A linear range from 5 to 250 ng/mL and from 5 to 400 ng/mL has been successfully validated for afatinib and ibrutinib with high accuracy and precision using accuracy profile strategy. The method is sensitive with low LLOQ. To the best of our knowledge, it is the first time that such analytical protocol (including *μ*SPE, UPLC and a fully validation) is described for these two TKis and seems to be the first description of this approach for these drugs. It represents perhaps a valuable and cheaper methodology that can be implemented in routine therapeutic drug monitoring across more laboratories.

## Figures and Tables

**Table 1:** Validation data of linear regression of AFA and IBR in human plasma.

Analyte	Regression model	VS	Concentration (ng/mL)	Trueness	Precision	Accuracy	Extraction efficiency
				Relative bias (%)	Repeatability/ intermediate precision (RSD, %)	*β*-expectation lower and upper tolerance limits of the relative error (%)	Average recovery (%) (*n* = 12, each level)
AFA	1		5	−0.24	5.60/5.65	[−13.38; 12.90]	88.0 ± 2.2
	2		25	1.31	5.54/4.94	[−9.96; 12.58]	
	3		75	0.63	2.70/2.63	[−5.44; 6.71]	
	4		125	1.29	2.23/2.42	[−4.52; 7.09]	
	5		175	0.56	1.87/1.66	[−3.23; 4.36]	
	6		250	−0.25	1.63/1.91	[−4.77; 4.27]	
DICLO			250	Internal standard			91 ± 7

IBR	1		5	−1.21	5.83/5.22	[−12.96; 10.54]	93.0 ± 9.0
	2		75	7.63	1.96/1.89	[3.28; 11.98]	
	3		150	0.77	1.85/2.83	[−7.94; 9.48]	
	4		250	−0.47	1.77/3.47	[−12.89; 11.95]	
	5		350	0.43	2.02/3.48	[−11.94; 12.79]	
	6		400	−0.07	1.87/2.25	[−6.00; 5.85]	
DICLO			250	Internal standard			90 ± 8

*n*: number of repetitions.
